# Carbapenemases in Enterobacteria, Hong Kong, China, 2009

**DOI:** 10.3201/eid1701.101443

**Published:** 2011-01

**Authors:** Yiu-Wai Chu, Viola W.N. Tung, Terence K.M. Cheung, Man-Yu Chu, Naomi Cheng, Christopher Lai, Dominic N.C. Tsang, Janice Y.C. Lo

**Affiliations:** Author affiliations: Centre for Health Protection, Hong Kong Special Administrative Region, People’s Republic of China (Y.-W. Chu, V.W.N. Tung, T.K.M. Cheung, M.-Y. Chu, J.Y.C. Lo);; Queen Elizabeth Hospital, Hong Kong (N. Cheng, C. Lai, D.N.C. Tsang)

**Keywords:** NDM-1, IMI-3, IMP-4, metallo-β-lactamase, class A carbapenemase, bacteria, Hong Kong, enterobacteria, expedite, letter

**To the Editor:** Carbapenems are often the recommended treatment for serious infections caused by extended-spectrum β-lactamase–producing enterobacteria. However, enzyme-mediated carbapenem resistance is increasingly reported worldwide. Carbapenemases are represented by 3 molecular classes of β-lactamase: A, B, and D (*1*). The best known class A carbapenemase is *Klebsiella* *pneumoniae* carbapenemase (KPC); KPC-producing enterobacteria are responsible for many hospital outbreaks. Class B carbapenemases are metallo-β-lactamases (MBL), which have the widest substrate spectrum. Class D OXA-type carbapenemases are found mainly in nonfermenting bacteria, except for OXA-48, which has been found only in enterobacteria.

In Hong Kong Special Administrative Region, People’s Republic of China, the Public Health Laboratory Centre routinely provides microbiological diagnostic services for government outpatient clinics and confirms the identity of bacterial isolates referred by other clinical laboratories. In 2009, among 18 enterobacteria isolates determined to be not susceptible to carbapenem, only 4 isolates—*Citrobacter freundii*, *Enterobacter cloacae*, *Escherichia coli*, and *K. pneumoniae*—were confirmed to produce carbapenemase. The *E. coli* isolate was from a government outpatient clinic; the others were from a regional hospital laboratory (Table).

For all 4 isolates, the modified Hodge test (*2*) demonstrated enzyme activity against ertapenem and meropenem. Previously described PCR and sequencing methods (*1*) identified the MBL IMP-4 in the *C. freundii* and *K. pneumoniae* isolates; the *C. freundii* isolate also possessed extended-spectrum β-lactamase CTX-M-9. The *E. coli* isolate harbored the recently described MBL called New Delhi metallo-β-lactamase (NDM-1) (GenBank accession no. FN396876) from India (*3*). The *E. cloacae* isolate possessed a class A carbapenemase IMI-like (Nmc-type) gene, and DNA sequencing confirmed its 97.2% nt and 97.6% aa identity to IMI-1. This IMI allele was subsequently designated IMI-3 (GenBank accession no. GU015024). For all 4 enterobacteria isolates, PCR was negative for OXA-48.

MIC determination by Etest and VITEK 2 (bioMérieux, Marcy l’Etoile, France) showed that all 4 isolates were resistant to ampicillin, amoxicillin/clavulanate, piperacillin/tazobactam, cefoxitin, cefuroxime, cefotaxime, and ceftazidime, according to Clinical and Laboratory Standards Institute breakpoints (*2*). Because IMI-1 was inhibited by clavulanate and tazobactam, the corresponding resistance in the IMI-3 positive *E. cloacae* isolate might result from other mechanisms, possibly AmpC β-lactamase, although PCR results for common AmpC alleles were negative (*4*).

All 4 isolates showed resistance to all 3 carbapenems according to the Clinical and Laboratory Standards Institute MIC criteria updated in June 2010 (Table), except for the NDM-1 positive *E. coli* isolate, which had an intermediate MIC for meropenem of 2 μg/mL. The IMP-4 positive *C. freundii* and *K. pneumoniae* isolates also seemed to be more multidrug resistant; they were resistant to nalidixic acid, ciprofloxacin, nitrofurantoin, and co-trimoxazole and susceptible to only amikacin and gentamicin. Conversely, the 2 organisms harboring IMI-3 and NDM-1 were susceptible to all these agents except for the NDM-1–positive *E. coli*, which was resistant to amikacin and gentamicin.

IMP-4 in *Acinetobacter* was first described in 2001 in a teaching hospital in Hong Kong (*5*). Since then, IMP-4 has been detected in several enterobacteria from mainland China and Australia. IMP-4 has spread throughout Hong Kong, crossing geographic and genus barriers; other new carbapenemases are also emerging. The association of IMP-4 with integrons and conjugative plasmids has been documented and possibly contributed to its propensity to spread. IMI-1 in *E. cloacae* was originally described in the United States in 1996. In 2005, IMI-2 (99% aa identity to IMI-1) in *Enterobacter asburiae* isolated from rivers in the United States was reported (*6*), and in 2006, a blood culture *E. cloacae* was found to possess IMI-2 in Hangzhou, China (*7*).

We report IMI-3 (aa identity 97.6% to IMI-2) in a urine isolate of *E. cloacae*, possibly a colonizer rather than the causative agent of the urinary tract infection because the urine specimen did not contain any leukocytes. The 2 IMP-4–positive enterobacteria isolates were also only transiently present; repeated cultures did not yield any carbapenem-resistant organisms despite the patients not having received any targeted therapy. Nonetheless, the presence of these transferable resistance determinants among patients with prolonged hospitalization is cause for concern. The NDM-1–positive *E. coli* isolate came from an outpatient of Indian ethnicity, who had hypertension, diabetes, and a urinary tract infection that responded to ciprofloxacin. This isolate was thought to have originated from the Indian subcontinent, where the patient had spent 3 weeks in March 2009; he had not been hospitalized in India. A similar case of travel-related NDM-1–positive *E. coli* isolated from urine has also been recently reported in Australia (8).

NDM-1 has the potential to be a worldwide public health problem (*9*). Our findings highlight the threat of carbapenemase-mediated resistance. Scrupulous surveillance must be maintained, and clinical microbiology laboratories should have adequate knowledge and capacity to identify these resistance determinants. To control the dissemination of these resistance determinants, coordinated infection control responses are needed at local, national, and international levels (*10*).

**Table:** Antimicrobial susceptibility results and ESBL detected for 4 carbapenemase-harboring enterobacteria isolates, Hong Kong, 2009*

Organism	Patient age, y/ sex	Patient location	Specimen	MIC, μg/mL (CLSI breakpoint for resistance)†									ESBL/ carbapenemase detected
				IMP (>4)	MEM (>4)	ERT (>1)	NA (>32)	CIP (>4)	NIT (>128)	AK (>64)	GN (>16)	SXT (>80)	
*Citrobacter freundii*	69/M	Hospital	Sputum	8	>16	>8	>32	>4	128	<2	8	>320	IMP-4, CTX-M-9
*Klebsiella pneumoniae*	60/M	Hospital	Bedsore	>16	>16	>8	>32	>4	>512	16	<1	>320	IMP-4
*Enterobacter cloacae*	68/F	Hospital	Urine	>16	>16	>8	4	<0.25	64	<2	<1	<20	IMI-3
*Escherichia coli*	64/M	Outpatient clinic	Urine	4	2	4	<2	<0.25	<16	>256	>16	<20	NDM-1

*ESBL, extended-spectrum β-lactamase; IMP, imipenem; MEM, meropenem; ERT, ertapenem; NA, nalidixic acid; CIP, ciprofloxacin; NIT, nitrofurantoin; AK, amikacin; GN, gentamicin; SXT, co-trimoxazole; NDM-1, New Delhi metallo-β-lactamase. †CLSI, Clinical and Laboratory Standards Institute, updated June 2010.
